# The wider social environment and changes in self-reported quality of life in the transition from late childhood to early adolescence: a cohort study

**DOI:** 10.1186/1471-2458-6-133

**Published:** 2006-05-17

**Authors:** Marjan Drukker, Charles Kaplan, Josien Schneiders, Frans JM Feron, Jim van Os

**Affiliations:** 1 Department of Psychiatry and Neuropsychology, South Limburg Mental Health Research and Teaching Network, EURON, Maastricht University, Maastricht, The Netherlands; 2 Youth Health Care Division, Municipal Health Centre, Maastricht, The Netherlands; 3 Graduate School of Social Work, University of Houston, Houston, Texas, US; 4 Division of Psychological Medicine, Institute of Psychiatry, London, UK

## Abstract

**Background:**

Neighbourhood socioeconomic disadvantage and social capital have been associated with adolescent well-being, but the majority of studies were cross-sectional, and the time window over which the neighbourhood may impact on development is unknown. Therefore, the contribution of the neighbourhood environment to adolescents' quality of life and the course of these effects during the period of transition from childhood to early adolescence was examined.

**Methods:**

A cohort of adolescents living in Maastricht (The Netherlands), with a mean age of 11.2 years at baseline and of 13.5 years at follow-up was followed. Adolescents who responded both at baseline and at follow-up were included in the analysis (n = 475). Multilevel regression analyses estimated neighbourhood effects while controlling for individual-level effects. Neighbourhood-level socioeconomic and social capital variables, individual-level confounders, and baseline values of the outcome measures were included in the models.

**Results:**

None of the neighbourhood factors was associated with changes in general health or mental health over the two-year period. However, two-year exposure to greater disparity between individual level socioeconomic status on the one hand and neighbourhood level of socioeconomic status on the other (e.g. high socioeconomic status adolescents living in deprived neighbourhoods and vice versa) negatively impacted on self-esteem and satisfaction.

**Conclusion:**

The neighbourhood environment per se does not contribute to change in quality of life during the transition to early adolescence. However, adolescents living in families whose socioeconomic status deviates from the mean level of neighbourhood socioeconomic deprivation may be negatively affected.

## Background

Previous cross-sectional research has demonstrated associations between neighbourhood factors and adolescent well-being, mental health and smoking initiation [1-3]. However, in order to make causal inferences longitudinal studies are required. In addition, the time window over which neighbourhood impacts on development is unknown. Effects demonstrated in adolescence may be evidence of an exposure that originated in childhood. Alternatively, neighbourhood effects may impact cumulatively over the developmental course with effects increasing linearly with exposure time. Previously, a longitudinal study [4] reported increasing youth-reported behavioural problems (YSR) between the ages of 11 and 13 years in socioeconomically deprived neighbourhoods. Another cohort study also reported more behavioural problems in 5-to 11-year olds in disadvantaged or low collective efficacy neighbourhoods in a 2-year time period [5]. Furthermore, a retrospective case-control study showed that neighbourhood socioeconomic disadvantage (NSD) was associated with increased rates of children's mental health service use when neighbourhood social cohesion was low [6].

Several mechanisms have been proposed in explaining why the neighbourhood may be associated with various mental health outcomes. Social factors, physical aspects and poor provision of services in disadvantaged areas may cause and/or aggravate health problems, and/or there is selective migration or retention of persons with mental health problems in disadvantaged areas [7-9]. A specific effect of social control may be expected because if neighbours correct deviant behaviour in an early stage of childhood development, this collective intervention may directly prevent the children from developing behavioural problems as well as indirectly provide them with self-confidence and a sense of protection [3]. However, the only study reporting on this issue was cross-sectional [3].

The two neighbourhood constructs measuring the wider social environment are NSD and social capital; social cohesion is a dimension of social capital. NSD is a primary concept of the quality of neighbourhood social and structural environment. It is synonymous with neighbourhood poverty, and low neighbourhood socioeconomic status. It represents a different concept than individual-level socioeconomic status and can impact on all residents of a neighbourhood, both affluent and poor. Usually NSD is a summary score of a series of neighbourhood-level objective socioeconomic measures. On the other hand, the concept of neighbourhood social capital can be best measured by asking community members; they are the best informants of their neighbourhood. Social capital can be seen as the "glue that holds society together" [10]. It has been defined as "those features of social organisations that act as resources for individuals and facilitate collective action, e.g. high levels of interpersonal trust and norms of mutual aid and reciprocity" [9,11,12]. Social capital is a characteristic that may apply to a group of persons living in the same state, country, or neighbourhood. In addition to these, many other groups can be defined to which the study of social capital may be relevant. However, the impact of social capital may differ depending on the level of aggregation. For example, acquaintances on the other side of the city or the country can be helpful finding a job, but this is rather similar with the effect of individual-level personal social networks. On the other hand, norms, values, and behaviour of neighbours can impact on every day life. Therefore, we specifically studied neighbourhood-level social capital. When restricting the definition of social capital to the neighbourhood-level, this is synonymous with the availability of social resources, social support and social control that neighbourhood residents can rely on [13]. Sampson and colleagues developed two collective efficacy scales that can be seen as constructs of social capital: (1) informal social control and (2) social cohesion and trust [14,15]. The first scale measures the willingness of neighbours to intervene in hypothetical neighbourhood-threatening situations, for example in the case of children misbehaving. The second scale measures bonds and trust among the residents of the neighbourhood. These two scales were used as measures of social capital in the present study.

Associations between NSD and various health outcomes have been widely studied in children and adolescents [1]. However, although neighbourhood social capital has been identified as an important contextual factor in adults' health, [9,10] the number of studies on neighbourhood-level social capital and adolescents' mental health is limited [3,16], and longitudinal studies on this issue are scarce [17].

Baseline results of the cohort used in the current study showed that NSD and social capital were associated with children's general health and satisfaction [3]. Children's mental health was specifically associated with one aspect of social capital: the degree of informal social control [3]. Because independence and autonomy increase during adolescence with more time spent away from the parents, increasing neighbourhood influences and decreasing family influences may be expected [18,19]. Early adolescence (11–15 years) involves many life events and developmental tasks, such as changing school and joining (and leaving) multiple peer groups, which may result in larger variability in changes in quality of life over time. We, therefore, hypothesised that associations between neighbourhood environment and health related quality of life would not be static, and that changes in quality of life from age 11 years into early adolescence would be similarly linked to the neighbourhood environment.

Previous research implied the need to account for several interaction effects. Firstly, effects of NSD could be different for families of high and low socioeconomic status [3,20]. Secondly, there may be an interaction between NSD and social cohesion and trust. Previous research showed that higher levels of social cohesion and trust mitigated the detrimental effects of NSD [6]. Another study indicated that social capital was associated with lower levels of behavioural problems, but only in affluent neighbourhoods [5].

Thus, the present paper studies associations between neighbourhood environment and changes in health related quality of life between late childhood and early adolescence, also considering interaction of neighbourhood variables with family socioeconomic status and interaction between NSD and social cohesion and trust.

## Methods

### Research design

Maastricht is a small city located in the extreme south of the Netherlands (122 000 inhabitants) with a predominantly white population. The present study aimed to follow a cohort of 1007 adolescents living in the 36 Maastricht residential neighbourhoods and attending the same grade [3]. At baseline and follow-up, adolescents and their parents were asked to fill in a quality of life questionnaire that also included individual and family confounding variables. All adolescents meeting inclusion criteria, rather than baseline responders only, were asked to fill-in the follow-up questionnaire. The present paper reports on the follow-up measurement two to three years after baseline (2002/2003).

The study was been approved by the Medical Ethics Committee of the Maastricht University Hospital (AZM). Both at baseline and at follow up, parents were asked to sign an informed consent form conform legal requirements in The Netherlands.

### Neighbourhood variables

The measure of NSD was based on various neighbourhood socioeconomic characteristics obtained from the Maastricht Statistics Department and Statistics Netherlands (CBS) [3]. To summarise these data an exploratory factor analysis (principal factors without rotation) was carried out. Two identified factors explained 70.0% of the total variance [3]. Percentage single parent families, ethnicity, non-voters, unemployment, unemployment more than 1 year, social security, social security more than 3 years, mean income, mean income for persons employed 52 weeks a year, percentages high and low incomes, and percentage economically inactive loaded on the first factor NSD. Single persons and various mobility variables loaded on 'residential instability'. Regression factor scores were calculated for NSD, yielding a continuous variable with mean 0 and unity standard deviation. Higher scores indicated more socioeconomic disadvantage. This variable had a normal distribution.

In order to assess social capital, approximately 200 adult inhabitants from each of the 36 Maastricht neighbourhoods, aged 20 to 65 years, were randomly selected, using the municipal database. Forty-eight percent of the 7236 selected adults responded (hereafter: community survey). These inhabitants received a questionnaire on social capital, which they were asked to send back. Social capital was measured using the two collective efficacy scales: informal social control (ISC) and social cohesion and trust (SC&T), developed by Sampson and colleagues [15]. The two sum scores were obtained from individual answers in the community survey and aggregated to the neighbourhood level [3]. The ISC scale measures the willingness to intervene in hypothetical neighbourhood-threatening situations, for example in the case of children misbehaving. The SC&T scale measures bonds and trust among neighbourhood residents. These data were collected separately and the neighbourhood-level variables can be matched with all Maastricht data collections, such as the family cohort studied in the present paper.

The validity of ISC and SC&T was demonstrated in a selection of Chicago neighbourhoods representing the whole city [14,15]. Collective efficacy was also associated with mortality in Hungary [21] and with child mental health in Chicago [22]. The construct and concurrent validity was also examined at the Maastricht study site: both informal social control and social cohesion and trust were highly associated with NSD [3] (construct validity) and with measures of social contacts and cosiness as described in [23] at the neighbourhood level (concurrent validity, unpublished results: Spearman's rho between 0.60 and 0.91, p < 0.001, n = 36). The concurrent validity of social cohesion and trust was also tested at the individual level (unpublished results: Spearman's rho 0.56 and 0.53, respectively, p < 0.001, n = 3047).

The three neighbourhood variables were standardized to mean zero and unity standard deviation. Higher scores indicated more NSD and lower levels of social capital [3].

### Adolescents' health-related quality of life

Both baseline and follow-up questionnaires of adolescents consisted mainly of the Child Health Questionnaire (CHQ) child form (87 items) [24]. The CHQ-subscales general health, mental health, and self-esteem were selected for the analyses [3]. Furthermore, items on satisfaction (including amongst others: satisfaction with relationships, with friends and teacher, school performance, leisure activities) were added to the questionnaire and a satisfaction scale was constructed combining self-esteem and satisfaction items [3]. Because this scale was constructed in the same way as the original CHQ scales [24], general health, mental health, self-esteem, and satisfaction scales could all range from 1 to 100 and were all positively scored, higher scores indicating better outcomes.

### Individual and family variables

Family socioeconomic status was assessed using occupational status and educational status. Occupational status was measured using the current or last profession of the parents, and scored according to the International SocioEconomic Index of occupational status ISEI-92 [25]. Parent questionnaires also assessed the highest level of completed education. Family occupational and educational status were based on the parent with the highest score. In order to ensure control for family level processes, a variable measuring the quality of child-parent interaction at baseline was included in the models as the family-level equivalent of neighbourhood social capital. This variable, parental perceived difficulty (in child raising), was measured using the NOSIK (Nijmegen Parental Stress Index Short Version), a Dutch 25 item questionnaire (items such as "I have much more problems raising my child than expected", and "I notice that I am less able to take care of my child than expected") [26]. Sum scores of the 11 items of the parent domain were used in the present analyses.

### Statistical analysis

All analyses were performed using STATA (version 7/SE) [27]. Hierarchically structured data were subjected to multilevel regression analysis [28] in order to investigate neighbourhood effects while controlling for individual effects. Multilevel or hierarchical linear regression techniques are a variant of the more often used unilevel linear regression analyses and are ideally suited for analysis of clustered data, in this case consisting of multiple persons clustered within a single neighbourhood. The βs are the regression outcomes of the predictors in the multilevel model and can be interpreted identically to the estimates in the unilevel analyses.

Regression models analysing quality of life (general health, mental health, self-esteem, or satisfaction) included baseline quality of life, parental occupational status, parental educational status, parental welfare recipient status, single parent family status, child's gender, grade retention (age), and parental perceived difficulty (NOSIK). Occupational status, parental perceived difficulty, and parental educational status were entered as dummy variables in the equation, high occupational status, high educational status, and low perceived difficulty being the reference categories. The above-mentioned individual and family variables were all individual-level variables in the analysis because only one child per family was included in the cohort. Neighbourhood-level variables were NSD, ISC, and SC&T (included separately).

Two a priori interaction terms were added to the models: NSD * individual socioeconomic status and NSD * SC&T. When results suggested interaction, two methods were used to further clarify the dynamics of the interaction. (1) If one of the interacting variables was educational status, analyses were performed stratified by combined categories (university or higher vocational, higher secondary or intermediate vocational, lower secondary or elementary). (2) If SC&T was the interacting variable, the model including the interaction term was used to calculate effects of NSD for very low social cohesion and trust neighbourhoods (i.e. SC&T variable-2SD), low social cohesion and trust (i.e. SC&T variable-1SD), average social cohesion and trust (SC&T variable), high social cohesion and trust (SC&T variable+1SD), and very high social cohesion and trust (SC&T variable+2SD), respectively.

## Results

### Descriptives and correlations

Of the 1007 adolescents in the cohort, 598 responded at baseline (59%) and 703 (70%) at follow-up. Of all baseline respondents, 79% responded at follow-up and these adolescents were included in the present analysis (n = 475). In 94%, address or neighbourhood was the same as at baseline. Adolescents' mean age was 11.2 years at baseline and 13.5 years at follow-up, and 52% was female. Generally, follow-up scores on the quality of life variables were lower than baseline scores (table 1), but baseline and follow-up scores were highly correlated (table 2).

### Associations between neighbourhood factors and changes in general and mental health

Neither crude analyses nor analyses controlling for confounders (see statistical analyses) showed large or statistically significant associations between neighbourhood factors and changes in general or mental health (data not presented).

### Associations between neighbourhood variables and changes in self-esteem and satisfaction

Self-esteem models suggested interaction effects between (1) NSD and SC&T (p = 0.13) and (2) NSD and parental educational status (p = 0.11), although statistically imprecise by conventional alpha. Therefore, table 3 shows regression coefficients (βs) of NSD stratified by parental educational status. Regression coefficients were calculated for 5 different levels of SC&T in all 3 strata of parental educational status. NSD was associated with a statistically significant positive change in self-esteem in adolescents with lower educated parents. This association was stronger when adolescents lived in lower cohesion and trust neighbourhoods (table 3). Conversely, NSD was associated with a decrease in self-esteem in adolescents with higher educated parents. However, these latter associations were not statistically significant.

Additionally, models analysing satisfaction suggested an interaction between NSD and parental educational status (p = 0.12). Therefore, regression coefficients stratified by parental educational status are presented in table 4. Again, NSD was associated with lower levels satisfaction in adolescents with higher educated parents, after controlling for baseline values and other confounders (table 4). Thus, adolescents of higher educated parents reported lower levels of satisfaction when living in socioeconomically disadvantaged neighbourhoods as compared to those living in affluent neighbourhoods.

### Individual-level variables and outcomes

Baseline values of general health, mental health, self-esteem, and satisfaction were all highly associated with their respective follow-up variables. All quality of life outcomes were lower in girls and in families with more perceived difficulty. Adolescents from single parent families reported lower levels of general health, self-esteem, and satisfaction, after controlling for baseline values. Lower parental educational status was negatively associated with changes in general health, albeit non-linearly – and it was positively associated with changes in satisfaction (data not presented).

## Discussion

Results showed that neighbourhood factors did not predict changes in general health or mental health in the period of transition from late childhood to early adolescence. However, NSD was associated with a positive change in self-esteem and satisfaction in adolescents from lower educated parents, while it predicted a negative change in adolescents from higher educated parents.

The baseline measurement of the present study did show associations between neighbourhood factors and general and mental health [3], and a Chicago cohort study in children aged 5–11 year, using a 2-year follow-up period, also showed associations with mental health [22]. Therefore, any effect of the neighbourhood may be restricted to children aged 11 years or younger. Informal social control may prevent mental health problems in primary school children only, because older children spent more time outside the neighbourhood, for example in the neighbourhood of their school. This is also in agreement with a study in older adolescents and young adults (15–25 years) that did not find evidence for an association in Mexico [29]. An U.S. experimental study including children between 8 and 18 years old (Moving to Opportunity: MTO) showed that children that were randomly assigned to receive vouchers to move to a non-poor neighbourhood had higher levels of mental health; again greatest effects were found in the younger children, possibly because older children can travel back to their old neighbourhood [17].

However, in the present study, levels of self-esteem and satisfaction increased when family socioeconomic status and NSD concurred. This indicates that the neighbourhood also impacts on 13/14 year-olds. In a previous study, minority children living in a dissonant environment were reported to have lower levels of self-esteem than minority children from segregated but protected environments [30]. The current results suggest this type of contextual interaction may apply not only to ethnic group status, but also to socioeconomic status itself.

The increased self-esteem of adolescents from lower educated families in poor neighbourhoods may indicate the mediating effects of peer influence on self-reported quality of life. Much of the emphasis in social capital-related research concerning the transition from childhood to adolescence has been on the family and school social control processes as well as neighbourhood factors. However, it has also been recognized that peer influences in life course transitions cannot be ignored and the suggestion has been made that peer attachments may have a neutralizing influence on the informal social bonds formed in family and school [31]. Youth in poor neighbourhoods with relatively weaker school and family social bonds may be likely to associate with specific peer groups of adolescents with similar family backgrounds as has been suggested in the literature on selection and assortative pairing in adolescent behaviour [32,33]. To our knowledge, the present article is among the first that reports these specific statistical associations between neighbourhood deficit factors and positive psychological well-being factors in youth. These findings will have to be replicated in future studies. The hypothesis that needs to be investigated is that under the specific conditions of persistent poverty and lower levels of parental education, disadvantaged youth may be more likely to pair with others in youth peer groups that have compensatory functions for deficits in the neighbourhood, schools and at home. Under these concentrated disadvantaged conditions where low self-esteem might well be expected, the youth peer group intervenes to provide a countervailing force producing a strong identity and with it an unexpected heightened sense of self-esteem. This process has been extensively documented in studies of youth gangs that are especially prevalent in urban areas characterized by concentrated disadvantage, migration and residential instability [34-36]. Although similar gangs do not exist in a small European city, like Maastricht, current results suggest that psychological outcome and socioeconomic conditions are similar in Maastricht.

Furthermore, both the positive association in adolescents of lower educated parents and the negative association in adolescents of higher educated parents between NSD and self-esteem appeared stronger in neighbourhoods low in social cohesion and trust. Thus, strong cohesion and trust mitigated effects of non-concurring family socioeconomic status and NSD. This is in line with a previous study, showing a stronger association between NSD and children's mental health service use in neighbourhoods low in social cohesion and trust [6]. This previous study concluded that neighbour interplay reduced the association between neighbourhood poverty and mental health, therewith stressing the beneficial effects of social capital. This NSD * social cohesion and trust interaction found in the present paper also indicates that the associations between cohesion and self-esteem are strongest in affluent neighbourhoods, in particular in children of lower educated parents. Thus, although children of lower educated parents tend to do worse in these areas, social cohesion and trust seems to reduce deterioration of self-esteem. On the other hand, this association was weaker in poor neighbourhoods and not statistically significant. This is in agreement with a previous study reporting that "sense of community" is positively associated with behavioural problems in affluent, but not in poor neighbourhoods [5]. However, the conclusion that social capital is only beneficial in affluent neighbourhoods is not warranted because social cohesion and trust also mitigated the effects of neighbourhood poverty both in the present and in a previous study [6].

### Methodological issues

Social capital is an umbrella term including many different constructs [37]. Only two of these constructs were included in the present study (informal social control; social cohesion and trust). These two scales were selected because these were the best validated measures at the time and the scales were used in a large cohort study in Chicago, with a very similar design, so that we could compare effects [38]. It is possible that analysing other constructs would yield different results. These previous analyses also showed that in Maastricht, ethnicity is not associated with quality of life outcomes [38]. Therefore, ethnicity was not included as a confounder in the present analyses. Because the Maastricht population is predominantly white, it may not be possible to extrapolate findings to larger and ethnically more diverse cities. However, for European small cities and towns like Maastricht, that typically have a low proportion of non-Western immigrants [39], the results may provide a public health perspective. In addition, a study in a relatively homogeneous population avoids difficulties of studying ethnically heterogeneous populations, such as language problems and cultural differences. More research in ethnic minority groups and ethically diverse populations can give more insight in the external validity of the present findings. Results regarding self-esteem and satisfaction may be stronger in ethnically more diverse populations because minority children living in a dissonant environment were reported to have lower levels of self-esteem than minority children from segregated but protected environments [30] (see above).

The strength of the present study is its longitudinal design that enables the prospective investigation of changes in quality of life in the transition to early adolescence. Furthermore, a principle objective of our methodology was to examine effects of neighbourhood variables that were obtained independently of the responding adolescents. Because perceptions of social capital are always biased by individual mental health status, it is difficult to disentangle cause and effect. The purpose of studying more distal mechanisms constituting objective social capital was realized by measuring social capital scale items in a group of informants that was different than the cohort investigated [40]. However, although both ISC and SC&T were measured independently of the study sample, answers of all informants are coloured by their individual characteristics [41]. On the other hand, individual socioeconomic and demographic composition provide the basis for social interactions in a neighbourhood and, therefore, controlling for individual characteristics leads to over adjustment [41].

The present paper has some limitations. First, in a longitudinal study there is always loss to follow-up. Although 79% of the baseline responders also responded at follow-up, which is relatively high, parental educational status differed between those who dropped out after baseline and those who responded to the follow-up questionnaire (t-test, p = 0.01). However, it is unlikely that this impacted on the results, as results (table 3 and 4) were stratified by parental educational status because of interaction between individual and neighbourhood socioeconomic status. This selective non-response could have led to a decreased power in the stratum of adolescents with low parental education, but table 3 shows greatest effects in this group.

Second, the response rate in the social capital community survey in adults was only 48% [3]. However, the community sample respondents and the general population between 20 and 65 years of age do have similar distributions in age, gender and ethnicity. Furthermore, all respondents were considered to be "key" informants about their own neighbourhood, with the implicit assumption that responders gave the same information about the neighbourhood as the non-responders would have given. The validity of the sample might have been judged differently if the principle objective was to obtain information on the person, not his or her neighbourhood. Thus, this information is more or less independent of the response rate. In order to verify this assumption, we examined *post hoc *associations between ISC and SC&T collected in the family cohort (parents), and those collected in the community survey (reproducibility). Neighbourhood scores on ISC and SC&T based on these questionnaires were highly correlated.

Inclusion of educational status and occupational status guarantees satisfactory control for individual level socioeconomic status in the Netherlands [42]. However, the possibility remains that residual confounding may have lead to spurious results at the neighbourhood level, because of omitted variable biases [1]. Families moving into poor or not moving out of poor neighbourhoods may differ from their peers although equally poor or affluent (e.g. in motivation, literacy etc). Smoking and obesity are factors that are associated with neighbourhood of residence and can hypothetically influence health outcomes [43-45]. Because at baseline none of the adolescents smoked, post hoc we repeated the analyses including obesity only as an extra confounder. Results were very similar, but effects in self-esteem in the middle stratum of parental education were somewhat stronger and statistically significant. In addition, although physical activity may be associated with health and quality of life outcomes, this measure was not included in the present study. Physical activity may be more easily obtainable or attractive in advantaged neighbourhoods, because of the neighbourhood environment or the presence of better equipped facilities. Given the fact that adolescents' quality of life in advantaged neighbourhoods as a result of more and better sports facilities, controlling for physical activity would result in smaller effects. Therefore, it is highly unlikely that physical activity is the reason that we did not find an effect of neighbourhood variables on quality of life. On the other hand, the problem of unhealthy reductions in physical activity tends to increase only in late adolescence [46], while our study ended in young adolescence. In addition, it is not likely that it impacts our main finding: the interaction between individual socioeconomic status and neighbourhood socioeconomic disadvantage.

Furthermore, none of the models showed statistically significant variance at the neighbourhood level (σ_μ_^2^), and intra class correlations (ρ) were low. Theoretically, variance at each level warrants including that level in the analyses [28]. However, neighbourhood researchers tend to analyse neighbourhood effects, even when intra class correlations and neighbourhood variation are low, and it is generally held that this is warranted [47]. In addition, in line with low neighbourhood-level variance, results showed no main effects of any of the neighbourhood variables. This does not rule out hypothesized interaction effects: neighbourhood-level variables were associated with outcomes in subgroups of adolescents.

The main outcomes of our study were quality of life and mental health and, therefore, the CHQ was included in the research instruments. CHQ-subscales are all continuous variables. Some prefer the use of dichotomous health outcomes. However, dichotomization results in loss of information and was not necessary here. This could reduce the comparability of the results to studies that did use dichotomous outcomes, but in neighbourhood research both dichotomous and continuous outcomes have been studied. Although the CHQ is a comprehensive instrument, the number of items per psychological domain is relatively low compared to psychological questionnaires like the CBCL. Therefore, the questionnaire is suited for research in the general population. However, in order to enable studies to address multiple research questions after a single data collection, one may prefer to further reduce the number of items. For general health, there is a widely-used and validated one-item alternative: "How do you perceive your health?" (answers on a 5-item Likert type scale: 1 excellent, 2 very good, 3 good, 4 fair, or 5 poor) [38]. This question (in Dutch) as well as one question on psychological problems (yes/no) are included in a new Maastricht data collection. Both concurrent (e.g. with the strengths and difficulties questionnaire, SDQ) and predictive validity will be studied in the future, using, amongst others, matching procedures with the psychiatric case register that records psychiatric service consumption. More research is needed to find and validate one-item alternatives for the self-esteem and satisfaction questions.

Associations between NSD, informal social control, and social cohesion and trust were so strong that collinearity problems would likely have arisen had these three variables been entered jointly in one regression model. Therefore, all neighbourhood variables were entered in the models separately, except when analysing interaction effects between two neighbourhood variables.

Finally, a previous study in another Dutch city on changes in behavioural problems between the age of 11 and 13 years, showed a statistically significant association between NSD and only one of the six behaviour outcomes [4]. Because all changes in behaviour were in the expected direction, the authors proposed that a longer follow-up period could reveal statistically significant changes. Future data collections with longer follow up periods may reveal more associations between neighbourhood factors and changes in general and mental health, and associations with self-esteem and satisfaction (in subgroups) could be replicated.

## Conclusion

None of the neighbourhood variables were associated with (changes in) general health or mental health. However, the present study showed that NSD and social capital were associated with self-esteem and satisfaction in specific subgroups of adolescents. Neighbourhood dynamics seemed to have put adolescents with non-concurring family socioeconomic status at disadvantage. However, while results might suggest that further segregation of the neighbourhoods improves self-esteem and satisfaction in these adolescents, this must be weighed against a far more severe level of disadvantages, such as social isolation with the full range of negative outcomes that co-occur.

## Competing interests

The author(s) declare that they have no competing interests.

## Authors' contributions

MD coordinated data collection and data editing, performed all analyses and wrote the manuscript.

CK has been involved in drafting and revising of the manuscript; in particular sociological input.

JS has been involved in revising the manuscript and data analyses

FF helped with the data collection and revised the manuscript

JVO had the overall supervision; including data collection, analyses and manuscript preparation

All authors read and approved the final manuscript

## Pre-publication history

The pre-publication history for this paper can be accessed here:



## Figures and Tables

**Table 1:** Descriptives: quality-of-life of the study sample at baseline and at follow-up.

	quality-of-life			
	general health	mental health	self-esteem	satisfaction

Baseline				
N	472	472	473	474
mean	79.9	82.9	81.0	80.0
standard deviation	15.1	11.4	11.9	11.0
range	23.8 – 100	23.4 – 100	28.8 – 100	32.3 – 100

Follow-up				
N	475	475	474	475
mean	77.4	78.4	75.2	69.1
standard deviation	14.7	12.5	11.9	11.9
range	23.3 – 100	28.3 – 100	32.7 – 100	31.3 – 99.0

Change				
N	472	472	472	474
mean	-2.5	-4.5	-5.9	-10.8
standard deviation	15.5	13.4	12.6	12.6
range	-44.2 – 49.6	-54.7 – 51.6	-50.0 – 42.3	-43.8 – 29.2

**Table 2:** Pearson correlations between quality-of-life variables at baseline and at follow-up.

	quality-of-life at follow-up			
	general health	mental health	self-esteem	Satisfaction

quality-of-life at follow-up				
general health	1	0.49^a^	0.50^a^	0.44^a^
mental health		1	0.66^a^	0.60^a^
self-esteem			1	0.88^a^

quality-of-life at baseline				
general health	0.46^a^			
mental health		0.38^a^		
self-esteem			0.44^a^	
satisfaction				0.40^a^

^a ^p < 0.001

**Table 3:** Multilevel regression analysis: association between neighbourhood socioeconomic deprivation (NSD) and changes in self-esteem βs and 95% confidence intervals (CI)^a^

**Parental educational status: University or higher vocational (n = 173)**			
		β^b^	CI
very little cohesion and trust	NSD	-4.91	-13.9; 4.04
little cohesion and trust	NSD	-3.93	-11.7; 3.89
average cohesion and trust	NSD	-2.95	-10.1; 4.19
good cohesion and trust	NSD	-1.97	-9.00; 5.07
very good cohesion and trust	NSD	-0.99	-8.53; 6.56

**Parental educational status: Higher secondary or intermediate vocational (n = 128)**			

		β^b^	CI
very little cohesion and trust	NSD^c^	5.60	-2.16; 13.4
little cohesion and trust	NSD^c^	4.57	-2.40; 11.5
average cohesion and trust	NSD^c^	3.53	-3.38; 10.5
good cohesion and trust	NSD^c^	2.50	-5.14; 10.1
very good cohesion and trust	NSD^c^	1.46	-7.47; 10.4

**Parental educational status: Lower secondary or elementary (n = 118)**			

		β^b^	CI
very little cohesion and trust	NSD	9.85^d^	2.57; 17.1
little cohesion and trust	NSD	7.97^e^	1.79; 14.2
average cohesion and trust	NSD	6.09^e^	0.17; 12.0
good cohesion and trust	NSD	4.21	-2.38; 10.8
very good cohesion and trust	NSD	2.33	-5.64; 10.3

^a^Stratified by parental educational status and models analysed using 5 different levels of SC&T.

^b^βs of NSD controlled for all confounders (baseline values, welfare recipient status, parental occupational status (5 categories), parental educational status (6 categories), parental perceived difficulty in child raising (5 categories), single parent family, gender, grade retention).

^c^Grade retention dropped due to collinearity.

^d^p < 0.01.

^e ^p < 0.05

**Table 4:** Multilevel regression analysis: the association between neighbourhood socioeconomic deprivation (NSD) and changes in satisfaction βs and 95% confidence intervals (CI)^ab^

Parental educational status: University or higher vocational (n = 173)	β	CI
NSD	-2.20^c^	-4.41; -0.04
		
Parental educational status: Higher secondary or intermediate vocational (n = 128)		
NSD^d^	1.61	-0.73; 3.96
		
Parental educational status: Lower secondary or elementary (n = 118)		
NSD	0.98	-1.09; 3.05

^a^Stratified by parental educational status.

^b^βs of NSD controlled for all confounders (baseline values, welfare recipient status, parental occupational status (5 categories), parental educational status (6 categories), parental perceived difficulty in child raising (5 categories), single parent family, gender, grade retention).

^c^p < 0.05.

^d^Grade retention dropped due to collinearity.

## References

[B1] Leventhal T, Brooks Gunn J (2000). The neighborhoods they live in: The effects of neighborhood residence on child and adolescent outcomes. Psychological Bulletin.

[B2] Frohlich KL, Potvin L, Gauvin L, Chabot P (2002). Youth smoking initiation: disentangling context from composition. Health Place.

[B3] Drukker M, Kaplan CD, Feron FJM, Van Os J (2003). Children's health-related quality of life, neighbourhood socio-economic deprivation and social capital. A contextual analysis. Social Science and Medicine.

[B4] Schneiders J, Drukker M, Van der Ende J, Verhulst FC, Van Os J, Nicolson N (2003). Neighbourhood socioeconomic disadvantage and behavioural problems from late childhood into early adolescence. Journal of Epidemiology and Community Health.

[B5] Caughy MOB, O'Campo PJ, Muntaner C (2003). When being alone might be better: neighborhood poverty, social capital, and child mental health. Soc Sci Med.

[B6] Van der Linden J, Drukker M, Gunther N, Feron FJM, Van Os J (2003). Children's mental health service use neighbourhood socioeconomic deprivation and social capital. Social Psychiatry and Psychiatric Epidemiology.

[B7] Reijneveld SA, Schene AH (1998). Higher prevalence of mental disorders in socioeconomically deprived urban areas in The Netherlands: community or personal disadvantage?. J Epidemiol Community Health.

[B8] Rutter M (1981). The city and the child. Am J Orthopsychiatry.

[B9] Kawachi I, Kennedy BP, Wilkinson RG (1999). The society and population health reader - Income inequality and health. The society and population health reader.

[B10] McKenzie K, Whitley R, Weich S (2002). Social capital and mental health. British Journal of Psychiatry.

[B11] Putnam RD (1993). Making democracy work. Civic traditions in modern Italy.

[B12] Coleman JS, Coleman JS (1990). The Foundations of Social Theory.

[B13] Drukker M, Kaplan CD, Van Os J, Harpham T, McKenzie K (2006). Chapter 5: Social capital and quality of life and mental health in Maastricht, The Netherlands; the neighbourhood matters. Social capital and mental health.

[B14] Sampson RJ, Raudenbush SW, Earls F (1997). Neighborhoods and violent crime: a multilevel study of collective efficacy. Science.

[B15] Sampson RJ (1997). Collective regulation of adolescent misbehavior: Validation results from eighty Chicago neighborhoods. Journal of Adolescent Research.

[B16] Dorsey S, Forehand R (2003). The relation of social capital to psychosocial adjustment difficulties: the role of positive parenting and neighborhood dangerousness. Journal of Psychopathology and behavioral assesment.

[B17] Leventhal T, Brooks-Gunn J (2003). Moving to opportunity: an experimental study of neighborhood effects on mental health. Am J Public Health.

[B18] Allison KW, Burton L, Marshall S, Perez-Febles A, Yarrington J, Kirsh LB, Merriwether-DeVries C (1999). Life experiences among urban adolescents: examining the role of context. Child Dev.

[B19] Brooks-Gunn J, Duncan GJ, Klebanov PK, Sealand N (1993). Do neighborhoods influence child and adolescent development?. American Journal of Sociology.

[B20] Ecob R, Macintyre S (2000). Small area variations in health related behaviours; do these depend on the behaviour itself, its measurement, or on personal characteristics?. Health Place.

[B21] Skevington SM, Sartorius N, Amir M, The WHOQOL-Group (2004). Developing methods for assessing quality of life in different cultural settings, the history of WHOQOL instruments. Social Psychiatry and Psychiatric Epidemiology.

[B22] Xue Y, Leventhal T, Brooks-Gunn J, Earls FJ (2005). Neighborhood residence and mental health problems of 5- to 11-year-olds. Arch Gen Psychiatry.

[B23] Drukker M, Van Os J (2003). Mediators of neighbourhood socioeconomic deprivation and quality of life.. Social Psychiatry and Psychiatric Epidemiology.

[B24] Landgraf JM, Abetz L, Ware JEJ (1996). Child Health Questionnaire (CHQ) a users manual.

[B25] Ganzeboom HBG, De Graaf PM, Treiman DJ (1992). A standard international socio-economic index of occupational status. Social Science Research.

[B26] Brock AJLL, Vermulst AA, Gerris JRM, Abidin RR (1992). NOSI Nijmeegse Ouderlijke Stress index.

[B27] StataCorp. (2001). Stata Statistical Software.

[B28] Snijders T, Bosker R (1999). Multilevel analysis, an introduction to basic and advanced modeling.

[B29] Harpham T, Snoxell S, Grant E, Rodriguez C (2005). Common mental disorders in a young urban population in Colombia. Br J Psychiatry.

[B30] Garcia Coll C, Lamberty G, Jenkins R, McAdoo HP, Crnic K, Wasik BH, Vazquez Garcia H (1996). An integrative model for the study of developmental competencies in minority children. Child Dev.

[B31] Sampson RJ, Laub JH (1993). Crime in the Making: Pathways and Turning Points through Life.

[B32] Kandel DB (1985). On Processes of Peer Influences in Adolescent Drug Use: A Developmental Perspective .. Alcohol and Substance Abuse in Adolescence.

[B33] Simons RL, Stewart E, Gordon LC, Conger RD, Elder Jr GH (2002). A test of life course explanations for stability and change in antisocial behavior from adolescence to young adulthood. Criminology.

[B34] Anderson E (1999). Code of the Street.

[B35] Vigil JD (2002). A Rainbow of Gangs: Street Cultures in the Mega-City.

[B36] Vigil JD (1988). Group processes and street identity: Adolescent Chicano gang members.. Ethos.

[B37] Lochner K, Kawachi I, Kennedy BP (1999). Social capital; a guide to its measurement. Health and Place.

[B38] Drukker M, Buka SL, Kaplan CD, McKenzie K, Van Os J (2005). Social capital and children's general health in different sociocultural settings.. Social Science and Medicine.

[B39] CBS (Dutch National Statistics Institute) (2006). StatLine. http://statline.cbs.nl/.

[B40] Buka SL, Brennan RT, Rich-Edwards JW, Raudenbush SW, Earls F (2003). Neighborhood support and the birth weight of urban infants. Am J Epidemiol.

[B41] Subramanian SV, Lochner KA, Kawachi I (2003). Neighborhood differences in social capital: a compositional artifact or a contextual construct?. Health Place.

[B42] Van Berkel-Van Schaik AB, Tax B (1990). Naar een standaardoperationalisatie van sociaal-economische status voor epidemiologisch en sociaal medisch onderzoek (Towards a standard measurement of socioeconomic status in epidemiological and social medical research). Sociaal-economische gezondheidsverschillen.

[B43] Kawachi I, Kennedy BP, Glass R (1999). Social capital and self-rated health: a contextual analysis. Am J Public Health.

[B44] Greiner KA, Li C, Kawachi I, Hunt DC, Ahluwalia JS (2004). The relationships of social participation and community ratings to health and health behaviors in areas with high and low population density. Soc Sci Med.

[B45] Van Lenthe FJ, Mackenbach JP (2002). Neighbourhood deprivation and overweight: the GLOBE study. Int J Obes Relat Metab Disord.

[B46] Andersen RE, Crespo CJ, Bartlett SJ, Cheskin LJ, Pratt M (1998). Relationship of physical activity and television watching with body weight and level of fatness among children: results from the Third National Health and Nutrition Examination Survey. Jama.

[B47] Raudenbush SW, Sampson RJ, American Sociological Association (1999). Ecometrics: toward a science of assessing ecological settings, with application to the systematic social observation of neighborhoods. Sociological Methodology.

